# Perceptions and decision-making with regard to pregnancy among HIV positive women in rural Maputo Province, Mozambique – a qualitative study

**DOI:** 10.1186/s12905-018-0644-7

**Published:** 2018-10-11

**Authors:** Carlos Eduardo Cuinhane, Kristien Roelens, Christophe Vanroelen, Samuel Quive, Gily Coene

**Affiliations:** 1 Department of Sociology, Eduardo Mondlane University, Maputo, Mozambique; 2 Vrije Universiteit Brussel (Brussels University), RHEA, Centre for Research in Gender, Diversity and Intersectionality, Brussels, Belgium; 3 Department of Obstetrics & Gynaecology, Faculty of Medicine & Health Sciences, Ghent University, Ghent University Hospital, Ghent, Belgium; 4 Department of Sociology, Vrije Universiteit Brussel (Brussels University), Brussels, Belgium; 5 Department of Philosophy and Ethics, Vrije Universiteit Brussel (Brussels University), RHEA, Centre for Research in Gender, Diversity and Intersectionality, Brussels, Belgium

**Keywords:** Perceptions, Decision-making, Pregnancy, HIV positive women, Mozambique

## Abstract

**Background:**

In preventing the transfer of HIV to their children, the Ministry of Health in Mozambique recommends all couples follow medical advice prior to a pregnancy. However, little is known about how such women experience pregnancy, nor the values they adhere to when making childbearing decisions. This qualitative study explores perceptions and decision-making processes regarding pregnancy among HIV positive women in rural Maputo Province.

**Methods:**

In-depth interviews and five focus group discussions with fifty-nine women who had recently become mothers were carried out. In addition, six semi-structured interviews were held with maternity and child health nurses. The ethnographic methods employed here were guided by Bourdieu’s practice theory.

**Results:**

The study indicated that women often perceived pregnancy as a test of fertility and identity. It was not only viewed as a rite of passage from childhood to womanhood, but also as a duty for married women to have children. Most women did not follow recommended medical advice prior to gestation. This was primarily due to perceptions that decision-making about pregnancy was regarded as a private issue not requiring consultation with a healthcare provider. Additionally, stigmatisation of women living with HIV, lack of knowledge about the need to consult a healthcare provider prior to pregnancy, and unintended pregnancy due to inadequate use of contraceptive were crucial factors.

**Conclusion:**

Women’s experiences and decisions regarding pregnancy are more influenced by social and cultural norms than medical advice. Therefore, education concerning sexual and reproductive health in relation to HIV/AIDS and childbearing is recommended. In particular, we recommend maternal and child healthcare nurses need to be sensitive to women’s perceptions and the cultural context of maternity when providing information about sexual and reproductive health.

## Background

In seeking to prevent the transfer of HIV to their children, couples in Mozambique are encouraged by the Ministry of Health to follow medical advice in their family planning. Men and women are advised to have an HIV test prior to pregnancy [1]. Couples found to be HIV negative are advised about practical methods to prevent HIV infection such as fidelity and condom use in all sexual relations [2]. They are also requested to be routinely tested for HIV every six months and to consult a healthcare provider before pregnancy [3]. Conversely, couples found to be HIV positive who do not want to become pregnant are requested to adhere to life-long antiretroviral therapy (ART) regardless of their CD4^1^ cell count, [4] and to use dual contraceptive methods – combining condom use with injections, intra-uterine devices or pills [1, 2]. When HIV positive couples want to become pregnant, they are advised to share their intentions with a healthcare provider [1].

Highlighting the dangers of infection, the government ran campaigns to promote [5] HIV testing and treatment, [6] the use of contraceptives for all men and women regardless of their HIV status, and the avoidance of risky sexual behaviours [7]. Moreover, HIV counselling, testing and treatment and contraceptive methods are provided free in all healthcare facilities [6]. Despite these campaigns and the availability of HIV counselling and treatment, studies [8–11] revealing high instances of unintended pregnancy have shown that the adherence to the recommended medical advice has been weak [12, 13].

The extant literature also shows that instances of unintended pregnancy are associated with low use of modern contraceptives [10] as well as a lack of condom use [11, 13]. A Mozambican national survey on malaria and HIV conducted in 2015 indicated that only 27% of adult married women (15–49 years old) and 50% of adult non-married women used modern contraceptives [14]. The lack of modern contraceptive use is largely related to misconceptions about this mode of birth control [15, 16]. In addition, a large segment of the Mozambican population uses traditional birth control methods such as herbs, amulets and charms^2^ believing they prevent pregnancy [17].

Moreover, studies in sub-Saharan Africa [9, 18, 19], including Mozambique, [20–22] have documented a low take up of HIV testing and antiretroviral therapy. This has been attributed to variant factors: discrimination, nondisclosure of HIV status, misinformation, drug shortage, stigma [19, 23, 24], lack of information about the importance of adhering to antiretroviral therapy [22], financial and social obstacles [20], and patients’ religious beliefs [25]. In Mozambique, for example, most patients seek a traditional healer before consulting a healthcare provider [26]. Such barriers arguably prevent couples from having a natural pregnancy with a minimal risk of infection among serodiscordant^3^ couples [27] and prevent passing on HIV to their infants [27, 28].

Despite an extensive literature about people living with HIV, few studies [8, 12] have explored how HIV positive couples perceive, and experience, decision-making processes when planning to get pregnant. In Mozambique in particular, little is known about how HIV positive women view pregnancy, what values they adhere to when taking decisions about childbearing, and significantly, how Mozambican healthcare providers perceive and counsel women on such decisions.

This article explores the perceptions and decision-making of HIV positive women who have recently become mothers in rural Maputo province, Mozambique. The specific focus is on perceptions, knowledge, practices, and medical advice with regard to decision-making about pregnancy. Women’s perceptions about having children are analysed, along with their decision-making trajectories with regard to their pregnancies, including the dominant values that influence decisions about childbearing. Furthermore, perceptions and practices of healthcare providers regarding attitudes of HIV positive women are also explored.

In order to better understand HIV positive women’s decision-making on pregnancy, we used Bourdieu’s practice theory [29]. Bourdieu argued that human activities take place within differently structured social spaces, each governed by rules and norms about how to act and interact with each other. Applying this theory can explain that women’s perceptions and decision-making on pregnancy are influenced by both individual concerns and prevailing norms within different social spaces; in this instance, the healthcare system and the family/community. By revealing a more nuanced picture of the variant factors that underpin the decision-making process, the research can make a contribution to more comprehensive approaches seeking to prevent the mother-to-child transmission of HIV and the prevention of unintended pregnancies among women living with HIV.

## Methods

### Study design and study sites

This qualitative study is part of a broader research programme entitled *Perceptions and practices regarding pregnancy care and infant feeding among HIV positive women in rural Mozambique*. Applying a grounded theory approach, [30] the research was conducted in the Namaacha and Manhiça rural districts of the Maputo province, located in the south of Mozambique.

In 2017, the population of Namaacha district was 48,933 [31] and serviced by ten healthcare facilities. Approximately 208,466 inhabitants lived in Manhiça district in 2017 [31] with 14 healthcare facilities and one rural hospital. These investigation sites were relevant for this study because of having the highest prevalence of HIV/AIDS in the country – accounting for 26% of all pregnant women living with HIV [32].

### Recruitment of participants and data collection

Recruitment and interviews of study participants took place between January and March 2015 in six healthcare facilities that implemented a prevention of mother-to-child transmission of HIV (PMTCT) program. Three healthcare facilities were selected in each district; one located at the centre and two in neighbourhoods. The centre of the district is relatively urbanised, while the neighbourhood settings are more rural. These differences were considered for purposes of analysis.

The study applied purposive sampling to select both HIV positive women and maternal child health (MCH) nurses. A total of 59 HIV positive women who had given birth and were breastfeeding were selected. Twenty-five (25) were located in Namaacha district and thirty-four (34) in Manhiça district, and one MCH nurse was selected in each healthcare facility. These participants were especially important in accessing women’s decision-making practices regarding pregnancy.

In selecting participants, the main researcher had previously visited the selected healthcare facilities with the ethical clearance, the objectives, and the research design. The MCH nurses working at maternal and child health service were introduced to the main researcher by the director of each healthcare facility.

In the central healthcare facilities, the maternal and child service is composed of four departments: antenatal, postnatal, a general paediatric department, and a child at risk clinic (CRC). There was one nurse in each department.

The healthcare facilities located in the more rural neighbourhoods had three departments: antenatal, postnatal and general paediatric departments. In these facilities, the general paediatric department offered services to all mothers and their infants regardless of their HIV status. It also served as a CRC. However, only one nurse was available to cover the three departments. In both facilities, a mother living with HIV had to first queue for postnatal service, secondly for general paediatric and lastly for the CRC. Nurses helped to identify the queues for mothers who were waiting to receive services at the CRC. Some mothers whose infants were less than one month old were assigned one appointment per week. Infants older than one month were assigned one appointment per month.

To access participants, all mothers at the CRC were approached, our identity was disclosed, and the objectives and benefits of the study were explained. Those who agreed to participate were interviewed. Of those who were approached, a total of eleven participants refused to participate. The most common reason cited was an unwillingness to share their experience of previous pregnancy and childcare.

Participants chose the place and time for the interview. The majority chose to be interviewed in the healthcare facility, the remainder made appointments at their homes. Interviews held in the healthcare facility took place under a tree, a convenient distance from the clinic consultation space, after the participants had attended all their consultations. The interviews lasted between 50 and 60 min. An audio recording was made following participants’ approval; some participants (*n* = 20) were uncomfortable with this technique. In all, 59 in-depth interviews were performed. The interviews ceased when saturation [33] was achieved.

The majority of participants were between 18 and 34 years, married or living with a partner. Most participants were farmers and had more than one child. Some participants lacked formal education (Table 1). The study also included five focus group discussions (FGDs) with HIV positive women. Three were conducted in Manhiça district and two in Namaacha district. Participants of these FGDs were recruited at the CRC. Each FGD lasted 60–90 min with 6–10 participants.

**Table 1:** Demographic characteristics of the study participants

Characteristics of participants	Manhiça (*n* = 34)	Namaacha (*n* = 25)	Total
	n/%	n/%	n/%
Age range			
18–24	14 (41.2)	10 (40.0)	24 (40.7)
25–34	17 (50)	11 (44.0)	28 (47.5)
35–39	3 (8.8)	4 (16.0)	7 (11.8)
Educational Level			
None	10 (29.4)	4 (16.0)	14 (23.7)
Less than primary education (1–6 years)	8 (23.5)	6 (24.0)	14 (23.7)
Primary education completed or more (7–9 years)	15 (44.1)	12 (48.0)	27 (45.8)
Secondary education completed (12 years)	1 (2.9)	3 (12.0)	4 (6.8)
Marital Status			
Single	5 (14.7)	7 (28.0)	12 (20.3)
Married/Living with a partner	27 (79.4)	17 (68.0)	44 (74.6)
Divorced/Widow	2 (5.9)	1 (4.0)	3 (5.1)
Number of Children			
1–2	20 (58.8)	14 (56.0)	34 (57.6)
3–4	11 (32.4)	7 (28.0)	18 (30.5)
5–6	3 (8.8)	4 (16.0)	7 (11.8)
Occupation			
Factor worker	0	1 (4.0)	1 (1.7)
Farmer	24 (70.6)	19 (76.0)	43 (72.9)
Housemaid	2 (5.9)	1 (4.0)	3 (5.1)
Own business/sale	6 (17.6)	1 (4.0)	7 (11.8)
Student	2 (5.9)	1 (4.0)	3 (5.1)
Teacher	0	2 (8.0)	2 (3.4)

The inclusion criteria for both participants in individual interviews and FGDs were: a) being 18–49 years old and living with HIV, b) having an infant between 0 and 2 years old, c) attending the CRC and d) agreeing to participate in the study. Both individual in-depth interviews and FGDs were conducted in Portuguese – the national language – for those who could read and write it. *Tsonga*, the local vernacular language, was used for those who could not understand Portuguese.

In addition, and after all interviews with HIV positive women had been undertaken, six semi-structured interviews with MCH nurses working in the PMTCT program were conducted. To access these participants, we selected MCH nurses working in the CRC. The objectives of the study were explained and interviews were requested. Participants scheduled the interviews, which lasted approximately 45 min, in their offices at the healthcare facility. The inclusion criteria included: a) being a MCH nurse working in a CRC; and b) agreeing to participate in the study. MCH nurses were aged between 23 and 35 years old, married or living with a partner, and all had children. All MCH nurses had attended a maternal child nurse course at the National Institute of Health Sciences.

## Interview guide

The in-depth interview, semi-structured interview, and FGD guides were developed and revised by all authors of the article, before a pilot test of the in-depth interview guide was carried out before data collection. After refining, the final guide was approved by all authors.

The in-depth interviews and the FGD guide explored similar topics: the experience of pregnancy; views about pregnancy after HIV diagnosis; HIV testing and experience of HIV treatment; experience of modern contraceptive methods as well as procedures used to decide about childbearing. FGD’s guide focused on views about pregnancy, pregnancy planning and decision-making in addition to participants’ experience of HIV treatment. To prevent unnecessarily upsetting the participants, questions about their emotional response to being diagnosed with HIV were avoided, rather, participants were able to voluntarily narrate their experiences about diagnosis, antiretroviral therapy and the challenges they faced.

The semi-structured interview guide for nurses consisted of exploring their practices and perceptions about women’s willingness to test for HIV as well as their adherence to treatment and contraceptive use prior to pregnancy.

### Procedures

This study obtained ethical clearance from the Faculty of Medicine of Eduardo Mondlane University and Maputo Central Hospital Bioethics committee, protocol number CIBS FM&HCM/73/2014. Verbal information in relation to the objective of the study was duly provided. Written consent was requested to each participant, so they could make an informed choice on whether or not to participate in the study. Those who could not read or sign were requested to choose someone in their trust to translate the information into the local language – *Tsonga* – and to sign on their behalf. All participants agreed to participate. Those who were able to write signed the informed consent forms. Three sociologists, who also spoke the local language, participated in the data collection: one was a male main researcher (the first author) and two female assistant researchers, who were trained on the objectives of the study and data collection instruments. The main researcher coordinated the fieldwork, interviewed the participants and also moderated the FGDs. Research assistants led interviews and conducted focus groups discussions.

### Data analysis

A thematic analysis approach [33, 34] was applied to obtain the key themes that emerged from the data, and following Bram and Clarke [34], this involved six stages. First, interviews were transcribed and then translated from Portuguese to English. Secondly, each transcription was read more than once and initial codes were generated [34]. Initial coding consisted in naming the narrative of the participants [33] and identifying interesting features for analysis [34]. This included experiences, practices and perceptions of the participants. in addition, frequently repeated words, or numbers emerging from participants’ narratives, were identified. Thirdly, the codes generated were used to identify and then search for various themes and sub-themes across the data [34]. In this sense, a theme is taken to be a set of words which captures something important about the data and represents a level of meaning expressed as a pattern within the dataset [34]. This process consisted of identifying themes within the explicit meaning of the data. Several themes were identified: women’s experiences of HIV testing and antiretroviral therapy prior to pregnancy; women’s practices during pregnancy; women’s perceptions and adherence to modern contraceptive use; and decision-making with regard to pregnancy. In each theme, several sub-themes were also identified. These included knowledge and practices of modern contraceptives, and the need for seeking a healthcare provider.

Fourthly, the identified themes were revised and refined as recommended by Bram and Clarke, [34]. All authors were involved in the process of reviewing and refining the themes. This consisted of checking the relationship between the coding and the themes generated, as well as evaluating the relationship between the sub-themes and themes. The final themes were then defined in the fifth stage [34] and presented in the findings section. At this point, we went back to the participants’ interviews and organised the narratives according to the identified themes. Content and supporting data of each theme were also identified.

The sixth and final stage of thematic analysis consisted of producing a report of these research outcomes. Moreover, a statistic package for Social Sciences (SPSS, version 23) was used to summarise the quantitative data emerging from the dataset.

## Results

This study explored several themes and sub-themes. Themes among HIV positive women included the following: HIV testing and adherence to antiretroviral therapy prior to pregnancy; HIV testing and adherence to antiretroviral therapy during pregnancy; perceptions of pregnancy; perceptions of risk of pregnancy after HIV diagnosis; experience of illness during pregnancy; decision for pregnancy; modern contraceptive use; and consulting a healthcare provider prior to pregnancy. In each theme, participants discussed their views about pregnancy and their adherence to medical advice recommended by the Mozambican Ministry of Health.

Themes among healthcare providers included counselling practices on HIV testing and antiretroviral therapy, and their perceptions of counselling women about pregnancy planning and adherence to medical advice prior to pregnancy. These themes enabled us to analyse what healthcare providers counselled to women and how they perceived women’s attitudes about medical advice.

## HIV test and adherence to antiretroviral therapy prior to pregnancy among HIV positive women

### HIV test prior to pregnancy

Most participants had undergone an HIV test prior to their last pregnancy and were HIV negative. The social characteristics of the participants, e.g. place of residence, age, level of education and marital status, influenced their willingness to have an HIV test prior to pregnancy. Participants living in Manhiça district, aged between 25 and 39 years old, with primary education and above, and who were married, were more likely to have an HIV test prior to pregnancy than other participants (Table 2). Those who already had children prior to the last pregnancy, reported having had an HIV test at the antenatal service, while others did the test simply to know their HIV status. However, the purpose of the test was not to plan their pregnancy. One participant said:

**Table 2:** Adherence of participants to HIV test prior to pregnancy

Characteristics of participants	Manhiça (*n* = 34)		Namaacha (*n* = 25)	
	HIV test prior to pregnancy		HIV test prior to pregnancy	
	Yes (n/%)	No (n/%)	Yes (n/%)	No (n/%)
Age range				
8–24	5 (14.7)	9 (26.5)	3 (12.0)	7 (28.0)
25–34	13 (38.2)	4 (11.8)	5 (20.0)	6 (24.0)
35–39	3 (8.8)	0	4 (16.0)	0
Educational Level				
None	5 (14.7)	5 (14.7)	1 (4.0)	3 (12.0)
Less than primary education (1–6 years)	6 (17.6)	2 (5.9)	3 (12.0)	3 (12.0)
Primary education completed or more (7–9 years)	9 (26.5)	6 (17.6)	6 (24.0)	6 (24.0)
Secondary education completed (12 years)	1 (2.9)	0	2 (8.0)	1 (4.0)
Marital Status				
Single	3 (8.8)	2 (5.9)	3 (12.0)	4 (16.0)
Married/Living with a partner	17 (50)	10 (29.4)	8 (32.0)	9 (36.0)
Divorced/Widow	1 (2.9)	1 (2.9)	1 (4.0)	0
Number of Children				
1–2	11 (32.4)	9 (26.5)	5 (20.0)	9 (36.0)
3–4	7 (20.6)	4 (11.8)	5 (20.0)	2 (8.0)
5–6	3 (8.8)	0	2 (8.0)	2 (8.0)
Occupation				
Factor worker	0	0	1 (4.0)	0
Farmer	15 (44.1)	9 (26.5)	10 (40.0)	9 (36.0)
Housemaid	1 (2.9)	1 (2.9)	0	1 (4.0)
Own business/sale	3 (8.8)	3 (8.8)	0	1 (4.0)
Student	2 (5.9)	0	0	1 (4.0)
Teacher	0	0	1 (4.0)	1 (4.0)
Knew HIV positive prior to pregnancy				
Yes	6 (17.6)	0	7 (28.0)	0
No	15 (44.1)	13 (38.2)	5 (20.0)	13 (52.0)

*I had an HIV test, but it was long time before I got married. I wanted to know my situation as I heard many people had HIV. Fortunately, I was negative. By that time, I was not planning to have a baby. I got married and I did another test when I was pregnant*. (Married, 1 child).

Participants who were not HIV tested prior to pregnancy disclosed that their pregnancies were not planned; as this participant explained:

*I did not do an HIV test because I did not plan to get pregnant. I did not think I could be HIV positive. I had never taken the HIV test before pregnancy*. (Single, 1 child).

Moreover, some participants lacked knowledge about the importance of having an HIV test before pregnancy on the basis that they did not perceive themselves as at risk. Thus, lack of knowledge and perceptions about their own health, militated against women seeking an HIV test prior to pregnancy – despite the risks of acquiring HIV being higher than they imagined.

### HIV status disclosure prior to pregnancy

Most participants who were HIV negative and married reported having disclosed this to their male partners. However, women who were HIV positive were less likely to disclose their HIV status due to fear of stigma and discrimination in the community. Participants did not disclose their HIV status to their male partners because they were afraid of rejection, abandonment, or loss of their homes.

*I am afraid to disclose my HIV positive status to my husband because he will not go to healthcare facility for HIV testing. I do not know what will happen to me if I disclose my HIV positive status. Besides, most men in this community* [Manhiça district] *reject and abandon their wives as soon they disclose their HIV positive status. I don’t want to lose my children and home*. (Married, 3 children).

The above narrative reveals how some participants face a dilemma when they are diagnosed HIV positive without involvement of their male partners. They often face the social stigma common to the environment or community. Notwithstanding this, many were unsure that their male partners would provide social support if they revealed their HIV status; they were aware of the experiences of other women who had disclosed their HIV positive status and consequently lost their children and homes. Taken together, these factors influenced participants decision not to reveal their HIV positive status. Nonetheless, participants who had the social support of their male partners, were more likely to disclose their HIV positive status. As a matter of fact, this study revealed that few participants who disclosed HIV positive status reported doing an HIV test together with their male partners.

*My husband knows that I live with this disease* [HIV] *because we went to the healthcare facility to do an HIV test together when I was pregnant for the first time.* (Married, 2 children).

### Adherence to antiretroviral therapy prior to pregnancy

Participants who were HIV positive prior to pregnancy did not often comply with antiretroviral therapy. Some participants cited lack of money for transport to go to the healthcare facility as a reason for not accessing antiretroviral drugs:

*I did not often take antiretroviral before pregnancy because I had no money for transport. The healthcare facility is a long way from my home and the transport is very expensive. There was a time when I would go one or two months without drugs because I had no money to pay for the transport, and the healthcare providers only give you antiretroviral drugs for one month.* (Married, 2 children).

With antiretroviral drugs being provided once a month, it was costly for some participants to travel. Some stated that they stopped taking them because they had not disclosed their HIV status, while others said that they did not really understand the benefits of antiretroviral therapy prior to their pregnancy. Moreover, participants who had prior experience with antiretroviral drugs and perceived that they were physically well, had eventually stopped HIV treatment. In addition, antiretroviral therapy was not perceived as necessary before pregnancy.

*I knew I was HIV positive in 2010. I started taking antiretroviral drugs at that time, but I stopped in 2012 because I was too tired taking these pills every day. Besides, at that time I was not pregnant and feeling very well. I did not know it was important to take antiretroviral drugs every day*. (Single, 1 child).

In the focus group discussions, most participants reported that they had abandoned antiretroviral therapy because of experiencing hunger after starting the treatment. Women said antiretroviral drugs were very strong, and when they are taken without enough food, they felt hunger and dizziness. Participants reported having problems with antiretroviral drugs because they did not have financial resources to buy food to support their subsistence. One of the participants narrated her experience as follows:

*When I started antiretroviral treatment, I felt hungry and dizzy shortly after taking the antiretroviral drugs because I did not have enough food. Sometimes, I would take tea as my main supper, on other days I would sleep without eating at all. But I still had to take antiretroviral drugs. I could not manage this situation because I had not money to buy food. So, I stopped taking antiretroviral drugs sometimes. I restarted antiretroviral therapy when I was pregnant.* (Married, 5 children, FGD).

## HIV test and adherence to antiretroviral therapy during pregnancy

### HIV test during pregnancy

The fact that the majority of participants learned of their HIV positive status at the first antenatal visit, shows that an HIV test had not been an aspect of their family planning. This was a frightening and stressful period for many. One participant recalled it as follows:

*I did not know I was HIV positive before the test at the antenatal care clinic. When the nurse told me about it, I was shocked and scared. I was very sad because I did not expect this result.* (Married, 1 child).

Almost all participants, including those who already knew their HIV positive status, reported having a test for HIV at their first antenatal care appointment. The majority of participants perceived that having an HIV test at the first antenatal care was a mandatory requirement. Moreover, they did not disclose their HIV status to the healthcare providers when they were requested to do a HIV test. Only one participant was not tested because she disclosed her HIV positive status to the nurses; as she explained:

*I did not do an HIV test at the antenatal care because I already knew I was HIV positive. When the nurse wanted to do it, I told her I was HIV positive and I showed her the documents showing that I was already taking antiretroviral drugs.* (Single, 1 child).

### HIV status disclosure during pregnancy

Most participants did not disclose their HIV positive status to their male partners after receiving their results. Some said they were not psychologically prepared, while others feared stigma and discrimination. They preferred their male partners to find it out for themselves at the healthcare facility. Participants reported giving letters of invitation from the healthcare facility to their male partners, but most of them said they would not go. This lack of male partner involvement in HIV testing prevented most participants from disclosing their HIV positive status. As one participant put it:

*No, I have not yet disclosed my HIV positive status to my husband because when I gave him the letter from health facility he read and kept it. He often says he has no time due to work. He has never been to the health facility for an HIV test. I do not know what he will say or do when I disclose my HIV status. It is better he goes for the HIV test and find out for himself*. (Married, 1 child).

Few participants who disclosed their HIV positive status said their male partners accepted the invitation letter from the healthcare facility to have an HIV test together. This shows that male partner involvement in HIV testing is a significant factor in HIV disclosure; as one participant recalled:

*The nurses gave me a letter inviting my husband to the healthcare facility. I gave him the letter and some days later, we went to the healthcare facility and we did the HIV test together. I repeated the test. It was found that we were both HIV positive, and the nurse explained us how to follow treatment.* (Married, 5 children).

### Adherence to antiretroviral therapy during pregnancy

Most participants reported that they started using antiretroviral drugs and took them consistently following their first antenatal visit. They reported that compliance with the medication regime was primarily to prevent HIV transmission to the baby as much as for their own health.

*In the beginning, I felt it was very complicated to follow the treatment and take antiretroviral drugs every day. Nurses advised me to do this in order to prevent HIV infection to my baby. I followed the treatment and I took antiretroviral drugs. Fortunately, my baby was born HIV negative (…). I am still following treatment for my baby and me.* (Married, 3 children).

Additionally, some participants perceived that treatment with antiretroviral drugs to prevent HIV infection to the baby during pregnancy, was mandatory; one participant explained.

*I took antiretroviral drugs during my pregnancy and I am still taking it to prevent HIV infection to my baby. The nurses told me that I have to take the medication to avoid passing the disease* [HIV] *to my child. I have no choice but to follow it.* (Single, 2 children).

This suggests that most participants perceived antiretroviral drugs as necessary only for preventing transmission from a mother to an infant. Similarly, most participants who were aware of their HIV positive status prior to their last pregnancy, did not comply adequately with antiretroviral therapy when they were not pregnant. Participants who had not disclosed their HIV positive status to their male partners said they also managed to comply with antiretroviral drugs throughout their pregnancy. Some reported their male partners were away from home – working in South Africa – while others told their male partners the medication was for the health of the baby.

*I take antiretroviral drugs with no problems because I tell my husband that the medication is for the health of the baby. Apart from antiretroviral drugs, nurses give us tablets for anaemia, malaria and pills to prevent pregnancy. My husband never checks the medication I bring from the healthcare facility, and he has never consented to go to the healthcare facility with me*. (Married, 4 children).

Although the above narratives show that participants who did not disclose HIV positive status to their male partners managed to take antiretroviral drugs, and therefore reduced the risk of passing HIV to the unborn infant, such a practice is not often safe. Participants may be prevented from continuing with antiretroviral therapy if their partners become aware of it. Nonetheless, the fact that many male partners worked far from home, lacked knowledge about HIV/AIDS, and were not involved in maternal and child healthcare, enabled HIV positive women to follow antiretroviral therapy during pregnancy.

## Perception of pregnancy among HIV positive women

Participants held mixed perceptions. For some, pregnancy was perceived as a test of fertility and a rite of passage from childhood to womanhood. Becoming pregnant certifies that one can bear children – to “*have children*”, to “*become adult*” and “*a mother*”. One participant explained this as follows:

*Pregnancy means that a woman is already adult and is responsible for taking care of her child. A woman is no longer a child. She becomes an adult and responsible for taking care of the child that will be born*. (Single, 1 child).

For others, pregnancy was viewed as part of women’s identity, as the duty of marriage, and as a way of achieving social status. Pregnancy relates, according to some participants, “*to being a woman*”, “*to becoming important*” and “*to fulfilling one’s responsibility as a spouse*”. Participants illustrated their views thus:

*Pregnancy means I am a woman. Every woman is supposed to become pregnant if she has a husband. When a woman is bearing children, she is fulfilling her task as a spouse*. (Married, 1 child).

*Pregnancy means that I am a woman that bears children and that I dignify and expand the surname of my husband’s family. I also contribute to the development of the country because I bear children to the family where I am married to. These children will grow up, work and contribute to the wellbeing of the country*. (Married, 5 children).

The data however, shows that the perceptions of participants vary according to social characteristics such as age, relational status and knowledge of HIV positive status prior to pregnancy. Younger women (18–34 years) perceived pregnancy as a test of fertility and as a rite of passage from childhood to motherhood. Older women (35–39 years) understood pregnancy as a means to achieve social status.

Women, who knew their HIV positive status and not yet had a child, also viewed pregnancy as a test of fertility. When they did get pregnant, they were happy because they had fulfilled their desire of becoming a mother like other women. As one of the participants revealed:

*When I knew I was pregnant I was very happy because I tried to get pregnant several times, but I could not conceive. When it was finally confirmed, it was the best moment of my life because I was going to have a child. It meant that I could bear children and have a family*. (Married, 1 child).

However, there were some women who were aware of their HIV positive status, had more than one child, and viewed pregnancy as both a burden to their health and their family finances. One participant explained her concerns:

*Pregnancy means to have children. But becoming pregnant nowadays does not help me a lot because we [my husband and I] are sick* [HIV positive]*. Apart from that, we also have to raise the children we already have. Although I felt good during pregnancy because I could not feel pain in my body, I did not want more children. But that is it. My husband wanted it and I became pregnant*. (Married, 3 children).

## Perception of risk of pregnancy after HIV diagnosis

Participants were familiar with the risks, and expressed their fear, of pregnancy. Some participants perceived pregnancy as an illness and recognised it could lead to death during gestation and/or childbirth. This view was based upon personal experience with a previous pregnancy; as one participant recalled:

*When I was pregnant, I was always sick and often not well... I started feeling unwell when I was 5 months pregnant and I only felt better after childbirth. I was afraid because I thought I could die during pregnancy or while giving birth*. (Married, 4 children).

Some participants harboured additional concerns about that they would transmit HIV to their babies, while others were anxious that they might die before their children reached the age of eighteen and that nobody would be there to take care of them.

*Being pregnant was complicated because besides managing my health, I was worried that the baby would be born with my disease* [HIV]*. I only felt happy when the baby was worn*. (Married, 3 children).

*When I knew I was HIV positive I was worried about my children. My parents have already died and I thought I could die and nobody would take care of my children. I live with my children and my husband. That worries me. I would like to be alive and see my children grow up to be eighteen years old when they can take care of themselves.* (Married, 4 children).

Participants who had not experienced sickness in their prior pregnancies viewed pregnancy as a normal condition. In the focus group discussions, participants considered it an illness only if a woman was always sick during her pregnancy.

*I would say pregnancy is normal when a woman does not get sick from the time she gets pregnant until childbirth. But, if she is often sick, then that is not normal. It is an illness because a woman is always in the healthcare facility to get treatment. For me, for example, pregnancy is normal because in my experience I have never been sick during pregnancy.* (Married, 3 children, FGD).

## Experience of illnesses during pregnancy among HIV positive women

Most participants experienced illnesses during pregnancy. The most common illnesses were constipation, fever, cough, headache, foot pain, stomach-ache, body pain, malaria, dizziness, vomiting, diarrhoea and vaginal wounds. Some participants associated the experienced illnesses with their HIV positive status. One participant reported her experience and views as follows:

*I got sick. I often felt pain in my body, was vomiting, and experienced diarrhoea. I think these were symptoms of the disease I have* [HIV]*. This is because since I started taking antiretroviral drugs, I no longer have such illnesses.* (Married, 2 children).

However, some reported the illnesses they experienced were unrelated to their health status because they were already taking antiretroviral drugs. They believed the reasons why women get sick during pregnancy were varied and not dependent upon their HIV positive status. One participant said:

*Before being pregnant I did not get sick, I was vomiting and feeling dizzy because I was pregnant. I was already taking antiretroviral drugs when I got pregnant. I know some women who were HIV negative and they often got sick when they were pregnant.* (Married, 3 children).

## The decision-making process with regard to pregnancy

### Planning pregnancy

In both districts, most participants stated that their pregnancies were not planned. Whether they were married or single, and regardless of knowledge of their HIV positive status, were more likely to have an unintended pregnancy. (Table 3). In addition, many participants aged between 35 and 39 did not plan their pregnancies. Several participants reported they unintentionally became pregnant because “*it appeared suddenly without expecting it*” or “*by chance*”. As one participant explained:

*Pregnancy is something that appears suddenly, even if you do not want it. As soon as you have a husband, you have to give him children.* (Married, 1 child).

**Table 3:** Adherence of participants to pregnancy planning

Characteristics of participants	Manhiça (*n* = 34)		Namaacha (*n* = 25)	
	Planned pregnancy		Planned pregnancy	
	Yes (n/%)	No (n/%)	Yes (n/%)	No (n/%)
Age range				
18–24	3 (8.8)	11 (32.4)	2 (8.0)	8 (32.0)
25–34	6 (17.6)	11 (32.4)	3 (12.0)	8 (32.0)
35–39	0	3 (8.8)	1 (4.0)	3 (12.0)
Educational Level				
None	4 (11.8)	6 (17.6)	0	4 (16.0)
Less than primary education (1–6 years)	2 (5.9)	6 (17.6)	2 (8.0)	4 (16.0)
Primary education completed or more (7–9 years)	2 (5.9)	13 (38.2)	1 (4.0)	11 (44.0)
Secondary education completed (12 years)	1 (2.9)	0	3 (12.0)	0
Marital Status				
Single	0	5	0	7
Married/Living with a partner	8 (23.5)	19 (55.9)	6 (24.0)	11 (44.0)
Divorced/Widow	1 (2.9)	1 (2.9	0	1 (4.0)
Number of Children				
1–2	7 (20.6)	13 (38.2)	4 (16.0)	10 (40.0)
3–4	2 (5.9)	9 (26.5)	2 (8.0)	5 (20.0)
5–6	0	3 (8.8)	0	4 (16.0)
Occupation				
Factor worker	0	0	0	1 (4.0)
Farmer	7 (20.6)	17 (50.0)	4 (16.0)	15
Housemaid	1 (2.9)	1 (2.9	0	1
Own business/sale	0	6 (17.6)	0	1
Student	1 (2.9)	1 (2.9	0	1
Teacher	0	0	2 (8.0)	0
Knew HIV positive status prior to pregnancy				
Yes	0	6 (17.6)	4 (16.0)	3 (12.0)
No	9 (26.5)	19 (55.9)	2 (8.0)	16 (64.0)

It is only when the data is broken down do different patterns emerge. Participants in both districts, aged between 25 and 34, who had completed secondary education, and had between 1 and 2 children, were more likely to have planned their pregnancies. Some married women who planned their pregnancy reported that it was the right time to have the first or another child; as one participant disclosed.

*I planned my last pregnancy because I wanted to have a third baby. The previous two have already grown up.* (Married, 3 children).

However, some respondents who were yet to have children, said they wanted to have a baby. One participant said:

*I became pregnant because I wanted a child. I wanted to be a mother. I was married, and I had no child.* (Married, 1 child).

Marriage then, for many of the participants, carried with it the expectation of having children. Once married, participants anticipated they would have children as a way of fulfilling familial and communal expectations. Some participants, for example, reported feeling pressured by family members, such as parents, because they were married but had not yet had a child. One participant recalled her experience as follows.

*When I became pregnant I felt good and emotional because in my family* [father’s family] *my sisters who were married already had a child. I was the only one who had not yet had a child. My family* [father’s family] *were already worried about why I could not get pregnant, so they* [father’s family] *were happy when they knew I was pregnant*. (Married, 1 child).

Such narratives about the social importance of infants in the community are congruent with participants’ perceptions about pregnancy. The desire to have a child is both an individual and a collective wish. It is reflected in the expectation that the importance of married women to bear children for her male partner’s family overrides many other factors and, shows how the social and cultural environment largely influence childbearing practices.

### Decision on pregnancy

There were variant reasons reported to pursue pregnancy. Some participants, reflecting the perception that adulthood is associated with both age and marital status, felt that they were now adult enough to properly care for children. The narratives strongly suggest that participants felt the need to have children was related to age and marital status.

*I decided to become pregnant myself because I was an adult and married. I was 30 years old and I had not a child yet*. (Married, 1 child).

Some participants decided to become pregnant unilaterally, while others said the decision was made with their spouse.

*My husband and I decided to have a baby. We talked about it and I felt I was ready to get pregnant.* (Married, 1 child).

For others, the husband made the decision, disregarding the will of the wife who presumably felt she was unable to refuse. One participant recalled.

*My husband decided that I should get pregnant. He wanted another child. We had one. This is the second child*. (Married, 2 children).

These narratives reveal that most women have very limited power to make decisions about becoming pregnant. This lack of autonomy certainly influenced the instances of unplanned pregnancy. Some women reported they became pregnant against their will, despite being HIV positive as one participant disclosed:

*I was not feeling very well when I became pregnant. I was scared because I did not want to become pregnant, as I already knew that I was sick* [HIV positive]. *Besides, we* [my husband and I] *were not* [doing] *very well in our relationship. But, when you become pregnant you cannot abort it*. (Divorced, 6 children).

Focus group discussions revealed that participants were less likely to take unilateral decisions. It was commonly perceived that women were obliged to bear their husband’s children and once they are pregnant they have no say in the future trajectory of the pregnancy. A participant puts it thus,

*This thing of deciding pregnancy is difficult. As long as you are married, and your child is grown up, you can get pregnant any time. Even if you get pregnant while you are still breastfeeding you cannot abort it. Once it happened to myself. I had to cease breastfeeding my baby at 8 months old because I was pregnant.* (Married, 3 children).

## Modern contraceptive use among HIV positive women

### Knowledge and use of modern contraceptives

All participants were aware of modern contraceptive methods: pills, injections, intra-uterine device (IUD) implants and male condoms. Some participants accessed information about them in the healthcare facility, while others got information in school and at other healthcare services. Participants said they received modern contraceptives at maternal and child health clinics. One participant explained it as follows.

*I know about tablets that prevent pregnancy. Nurses often talk about it during pregnancy and breastfeeding. You can choose to have pills or an injection. When you are pregnant they give and advise us to use a condom. They give us male condoms. Now that I am breastfeeding, they give me pills and male condoms.* (Married, 1 child).

However, other participants reported that they occasionally obtained pills from private pharmacies. This was common when participants finished their supply of pills and were not able to get to the healthcare facility. One participant reported.

*Sometimes I get pills from the private pharmacy near my home. This happens when I finish pills that the nurses give me and I have no time to go to the healthcare facility. Nurses give me pills for one month, meaning that I have to go to the healthcare facility every month to access it.* (Married, 2 children).

### Reasons to use modern contraceptives

Participants offered several reasons for using modern contraceptives. Some reported that they used pills prior to the decision to have a child.

*Before getting pregnant I was using pills to prevent pregnancy because I had not decided to get pregnant again. I stopped using pills when I wanted to get pregnant.* (Married, 4 children).

However, some participants who had disclosed their HIV positive status to their male partners reported using male condoms during the pregnancy and breastfeeding period. Condom use was associated with the fact that participants often perceived this practice as a way to prevent passing HIV to their infants.

*I started using a male condom when I was pregnant. My husband accepted it because the nurses advised a condom would prevent passing HIV infection from me to the baby*. (Married, 2 children).

*I use male condom now that I am breastfeeding. My husband accepts it. He knows I am HIV positive and I am taking antiretroviral drugs. I cannot do anything without informing him* [husband]. (Married, 1 child).

In addition, contraceptive methods such as injections were sometimes preferred. Participants used injections to prevent a repeat pregnancy while their babies were still young.

*I am using injections to prevent pregnancy because the baby is still only eight months years old. Nurses advised me to prevent pregnancy until the baby is 24 months old*. (Married, 1 child).

In focus group discussions, participants explained they used modern forms of contraception until their baby was between 18 and 24 months old, because it was only deemed acceptable to have another child after this amount of time had elapsed.

*Women must space pregnancies. For a woman to become pregnant, a child should have 18 months or 24 months. It is a responsibility because it is necessary to take care of the baby (…). Women should not become pregnant before a baby has grown up enough. It is bad because if a woman does not space pregnancies children will not grow healthy.* (Married, 2 children, FGD).

### Reason for not using modern contraceptives

The majority of participants did not use modern contraceptives consistently before pregnancy. Some said they were married and their child had grown up, while others stated their male partners were not at home – they were away working in South Africa. This practice contributed to unintended pregnancy as they could not take birth control pills when their male partners came back home.

*I do not use any contraceptive method because I am still breastfeeding (…). My husband works and lives in South Africa and comes home once a year, especially at the end of the year.* (Married, 1 child).

Regardless of any disclosure of their HIV status some said that their male partners refused to use modern contraceptives. The following narratives illustrate how women often had no power over the decision not to use contraceptives.

*My husband does not accept to use condoms (…). He does not allow me to take pills; he says these things are not useful*. (Married, 2 children).

*I was not using a condom during pregnancy because my husband does not accept it. Besides, I have not yet told him I am HIV positive*. (Married, 1 child).

Several single women said they did not often take birth control pills consistently – often forgetting to take them. This inconsistent use of contraceptives led to an unintended pregnancy, as one participant revealed.

*I used to take pills … but I think I forgot to take it some days… I got pregnant.* (Single, 1 child).

Some participants said they did not use modern contraceptives during the first six months of breastfeeding. They reported their mothers-in-law advised not to engage in sexual intercourse during this period because it would spoil breast milk and the baby would get sick.

*I do not use contraceptives to prevent pregnancy because I am still breastfeeding. My mother-in-law advised me not to engage in sexual activities before the baby completes six months. She said the baby will have diarrhoea or he will not grow up very well. He will be six months next month* [April 2015]. (Married, 2 children).

Although some participants abstained from sex during the first six months following childbirth, many reported having unprotected sexual intercourse after six months. Participants perceived that it was acceptable to resume sexual activities because the baby was “grown up”. They also said that their male partners assumed that after six months, sexual intercourse was no longer a danger to the baby.

*We have resumed sexual activities because the baby has grown up. He is 7 months now, but I am still breastfeeding. I do not use contraceptive and condom because my husband does not accept these things. He says sexual activity is no longer prohibited*. (Married, 1 child).

### Problems experienced during the use of modern contraceptives

Some participants experienced problems with contraceptive injections and pills. This included irregular menstruation when using these methods. These problems prevented some participants from consistently using contraceptives. One participant narrated her experience:

*I started using injections two months after childbirth. But I was not feeling well. I stayed two months without seeing menstruation. I decided to give up. Then, I changed to pills. But I am now having problems again. My menstruation has been coming out for one month now. It does not stop. I have not yet gone to the health facility to explain it*. (Mother, 2 months).

In addition, participants shared mixed experiences with regard to injections during focus group discussions. They felt that it was not necessarily a safe way to prevent pregnancy. Some participants reported getting pregnant while having injections. One participant explained it as follows.

*Sometimes the injection is not strong enough to prevent pregnancy. Maybe it depends from a person to person. I was applying injection, but after some time, I started feeling something in my belly. Four months later, I went to the healthcare facility and the nurses told me I was pregnant. How was I pregnant if I was taking injections?... I do not understand.* (Married, 4 children).

## Consulting a healthcare provider prior to pregnancy

### Knowledge and practice of seeking a health provider prior to pregnancy

Most participants lacked knowledge, or were not advised, of the need to consult a healthcare provider prior to pregnancy; as one participant explained.

*I did not know I had to consult a healthcare provider before pregnancy. I have never been advised about that*. (Widow, 1 child).

This was also common amongst participants who had two or more children and had knowledge of their HIV positive status. As a consequence, most did not seek a healthcare provider prior to pregnancy (Table 4). Those who did, wanted to know if their health status and level of CD4 cells would allow them to become pregnant, as one participant explained:

*I went to the healthcare facility to consult a healthcare provider before pregnancy because I know I am sick* [HIV positive]. *In my status, when CD4 is low it is not allowed to become pregnant. After analysing my situation, a healthcare provider advised me to become pregnant*. (Married, 3 children).

**Table 4:** Adherence of participants to health provider advice prior to pregnancy

Characteristics of participants	Manhiça (*n* = 34)		Namaacha (*n* = 25)	
	Consulted a health provider		Consulted a health provider	
	Yes (n/%)	No (n/%)	Yes (n/%)	No (n/%)
Age range				
18–24	1 (2.9)	13 (38.2)	0	10 (40.0)
25–34	2 (5.9)	15 (44.1)	2 (8.0)	9 (36.0)
35–39	1 (2.9)	2 (5.9)	1 (4.0)	3 (12.0)
Educational Level				
None	1 (2.9)	9 (26.5)	0	4 (16.0)
Less than primary education (1–6 years)	1 (2.9)	7 (20.6)	1 (4.0)	5 (20.0)
Primary education completed or more (7–9 years)	2 (5.9)	13 (38.2)	1 (4.0)	11 (44.0)
Secondary education completed (12 years)	0	1 (2.9)	1 (4.0)	2 (8.0)
Marital Status				
Single	1 (2.9)	4 (11.8)	1 (4.0)	6 (24.0)
Married/Living with a partner	3 (8.8)	24 (70.6)	2 (8.0)	15 (60.0)
Divorced/Widow	0	2 (5.9)	0	1 (4.0)
Number of Children				
1–2	3 (8.8)	17 (50.0)	1 (4.0)	13 (52.0)
2–4	1 (2.9)	10 (29.4)	2 (8.0)	5 (20.0)
5–6	0	3 (8.8)	0	4 (16.0)
Occupation				
Factor worker	0	0	0	1 (4.0)
Farmer	3 (8.8)	21 (61.8)	2 (8.0)	17 (68.0)
Housemaid	0	2 (5.9)	0	1 (4.0)
Own business/sale	1 (2.9)	5 (14.7)	0	1 (4.0)
Student	0	2 (5.9)	0	1 (4.0)
Teacher	0	0	1 (4.0)	1 (4.0)
Knew HIV positive status prior to pregnancy				
Yes	0	6 (17.6)	3 (12.0)	4 (16.0)
No	4 (11.8)	24 (70.6)	0	18 (72.0)

One participant consulted a family member who worked at the healthcare facility because she was experiencing a fertility problem and wanted tablets to address it. She recalled it and said:

*Yes, I consulted a nurse who is one of my family members. I wanted to know about tablets of fertilization because I could not get pregnant*. (Married, 1 child).

A few participants, who were HIV positive prior to pregnancy were aware of the advice to consult a healthcare provider. However, they believed there was no need. They perceived that only those who had problems of fertility ought to consult a healthcare provider prior to pregnancy, as the following narrative shows.

*My husband and I did not consult a healthcare provider before I got pregnant because we thought there was no need. I do not have a problem of fertility because this is my second child. Even with the first child we did not seek a healthcare provider before pregnancy*. (Married, 2 children).

Nonetheless, some did not seek a healthcare provider, even though they were experiencing fertility problems. Instead, they sought traditional advice and remedies in seeking to become pregnant. In most of these instances, the participants were not aware of their HIV positive status before the pregnancy. One participant stated:

*I heard that some women consult a healthcare provider to become pregnant, but I did not do so. I could not become pregnant for a long time, but I used only traditional remedies. After some time, I became pregnant*. (Married, 1 child).

### Perceptions regarding seeking a healthcare provider prior to pregnancy

Most participants said they would have consulted a healthcare provider before pregnancy if they were advised. However, some participants expressed their concern about the potential disclosure of their HIV positive status, and their anxieties over whether or not their male partners would accept it.

*For me, there is no problem to consult a healthcare provider before pregnancy. It is not difficult. But, the problem is with my husband. He does not know that I am HIV positive. I gave him the invitation letter when he came from South Africa, but he did not go for an HIV test yet. He kept it and whenever he comes back, he says he will go to the healthcare facility. Maybe that could make the process difficult*. (Married, 2 children).

Others said they were happy to consult a healthcare provider before pregnancy, but they viewed decision-making about pregnancy as a private matter. This perception, together with lack of knowledge can prevent participants from seeking a healthcare provider.

*If a healthcare provider advises me to consult him before pregnancy, I will do that. But deciding when to become pregnancy is my private issue*. (Married, 3 children).

In the focus group discussions, most participants agreed that pregnancy decision-making was a matter for a woman and her husband, especially when their current baby was two or more years old. Therefore, they felt that there was no need to consult a healthcare provider.

*To decide to become pregnant is an issue between my husband and me. I only get pregnant when I am happy and when I want. It is my option.* (Married, 4 children).

This reveals a distinct lack of fit between the role that the healthcare provider actually plays in giving advice and support in family planning, and the perception amongst participants that such institutions interfere in the private deliberations between couples over choosing when to have children.

## The practice of maternal and child health nurses when counselling on HIV tests and antiretroviral therapy

### The practices of maternal and child health nurses on counselling for HIV testing and antiretroviral therapy prior to pregnancy

Maternal and child health nurses reported low adherence to HIV testing among women prior to pregnancy. They said they often gave lectures about HIV prevention and advised all patients attending the healthcare facility to have an HIV test. However, such an effort did not translate to high adherence to HIV testing. One of the nurses explained:

*We often give lectures to all people who come to the healthcare facility for consultation. We talk about the different type of diseases, including HIV and AIDS. We talk about prevention methods and treatment. We also advise all participants to adhere to an HIV test*. (MCH nurse).

Despite this, maternal and child health nurses reported that almost no women sought an HIV test prior to pregnancy. One of the maternal and child nurses, who had worked in the department of counselling and voluntary testing, recalled her experience:

*Few people adhere to the HIV test. When I was working in the department of counselling and voluntary testing, I could stay one or two days without a single person requesting an HIV test. It is rare to see people coming voluntarily for an HIV test. I do not remember to have attended a woman who was looking for an HIV test because she was planning to become pregnant.* (MCH nurse).

In addition, maternal and child health nurses said most women who are tested HIV positive prior to pregnancy, tend to comply with antiretroviral therapy when they are physically ill. However, when they recover and feel physically healthy, they abandon HIV treatment. One maternal and child health nurse said:

*Some women know their HIV positive status before they get pregnant. They adhere to antiretroviral drugs mostly when they are sick. But, after they feel better, they abandon it. I have several experiences of women who abandoned antiretroviral therapy. When I meet them in the community, I talk to them about the importance of continuing with HIV treatment. Some say they are feeling physically good, and promise to come back, while others say they are continuing treatment in another healthcare facility*. (MCH nurse).

Other maternal and child health nurses reported that some women often went to the healthcare facility for antiretroviral drugs, but often neglected to take it at home.

*Sometimes, women follow HIV treatment and they pick antiretroviral drugs, but when they arrive at their homes they do not take it. We discover it when they become sick and we ask if they really take antiretroviral therapy regularly; and some reveal that* [they] *have some antiretroviral drugs kept at home. They did not take it as recommended*. (MCH nurse).

### Practices of maternal and child health nurses counselling on HIV test and antiretroviral therapy during pregnancy

Maternal and child health nurses reported that most women learned of their HIV positive status during pregnancy. They said all pregnant women who had been unaware of their HIV positive status are counselled and have to agree to HIV testing during pregnancy. They also said that HIV tests should not be repeated for pregnant women with known HIV positive status. However, they reported some women do not disclose their positive status at the antenatal care service, therefore they are tested as if it were the first time.

*All pregnant women have to do an HIV test on their first antenatal care visit. We counsel them, and we explain about the need of the test and its importance to their health and that of the baby. But, most women do not reveal their HIV positive status. In this case, we test them. Some already know their HIV positive status, but they do not believe it, and others do not reveal it because they have abandoned antiretroviral drugs. A woman who is taking antiretroviral drugs has documents that show she often complies with HIV treatment. Most women hide these documents when they come to the first antenatal care visit*. (MCH nurse).

Maternal and child health nurses reported that most patients tended to comply with antiretroviral therapy during pregnancy and while breastfeeding, however, they abandoned the treatment after weaning. Nurses said women often perceived HIV treatment as similar to pregnancy and breastfeeding, believing that antiretroviral therapy should only be resumed when they get pregnant again.

*Women comply with antiretroviral therapy during pregnancy and breastfeeding. But, once they stop breastfeeding and know the final result of the HIV status of their babies, most abandon HIV treatment. They most often restart it when they get pregnant again. Some even move to another healthcare facility when they get pregnant and when they arrive there, they do not disclose their HIV positive status*. (MCH nurse).

Maternal and child health nurses often attributed women’s poor adherence to antiretroviral therapy program to the non-disclosure of their positive status to male partners. They reported that letters of invitation for male partners to visit the healthcare facility were seldom responded to positively. One nurse explained,

*Every pregnant woman receives a letter of invitation after an HIV test regardless of the result. The letter requests a male partner to go to the healthcare facility to learn about the health of the foetus. We never mention an HIV test in the letter, but very few come here after receiving it. The majority do not come because they already know they will be requested to do an HIV test*. (MCH nurse).

Maternal and child health nurses said that HIV positive women were afraid to disclose their HIV positive status to their male partners due to fear of stigma and discrimination in the community. Some nurses reported HIV positive women and men preferred to adhere to HIV treatment in a healthcare facility located in a different community to avoid discrimination.

*Most women who test HIV positive say they are afraid to tell their husbands about it. This is the reason why we invite their husbands to the healthcare facility. Besides, I have witnessed that most women and men who live with HIV do not come to this healthcare facility. They go to another healthcare facility far from their community because they do not want to be seen by their neighbours and family members that they are receiving HIV treatment. They go to another healthcare facility where nobody knows them.* (MCH nurse).

## Practices and perceptions of maternal and child health nurses regarding counselling on pregnancy planning and adherence to medical advice prior to pregnancy

### Practices of maternal and child health nurses regarding women’s adherence to modern contraception

Maternal and child health nurses reported that women received and were advised to adhere to modern contraception before pregnancy and after childbirth. They said women requested a choice of contraceptive method from pills, injections and intra-uterine devices. Apart from these methods, they reported advising women to use a condom.

*We talk about modern contraceptives in every meeting with women; during pregnancy, prenatal, breastfeeding or other meetings. We advise women to choose to use pills, injections or intra-uterine device. We advise them to use contraceptives to prevent pregnancy. We tell women to avoid pregnancy before the baby completes 24 months. We also give, and teach them how to use, a male condom. When they are pregnant we advise them to use a condom. We give them contraceptives including male condoms every month when they come to the healthcare facility*. (MCH nurse).

Despite this, they reported that most women still fell pregnant before the advised minimum period of 24 months, largely due to a lack of compliance. This was strongly associated with male partners’ attitudes to modern forms of birth control, which were commonly perceived as encouraging infidelity among women. Women received male condoms at the healthcare facility, but they threw them away as their husbands never used them.

*When we give male condoms, women throw them away before they arrive at their homes. They often say their husbands do not accept to use condoms. They also say their husbands do not allow them to use contraceptives because they think their wives may engage into sexual activities with other men*. (MCH nurse).

As a means to help women comply with the use of contraceptives, maternal and child health nurses reported keeping women’s family planning cards^4^ at their offices and counselling the women’s partners about modern contraception. One nurse explained:

*I have many family planning cards in my drawer that women asked me to keep for them to avoid that their husbands discovering that they use contraceptive methods (…). I give them pills and they take it at home without disclosing to their partners. When their husbands do not see the family planning card, they have less chance to discover that their wives take pills.* (MCH nurse).

Another nurse reported helping women who had difficulties with their male partners regarding contraceptive use. She narrated how she managed such problems:

*Some women that use implants also get problems with their husbands. Once I fitted an implant to a woman after childbirth, and when her husband discovered* [it] *he told her to take it away. I invited her husband and I explained him that if his wife did not use an implant she would get pregnant before the baby was 2 months old and that would harm the baby. I told him I would take out the implant after the baby completed 24 months. After this explanation, he accepted my advice and only by this way the woman kept the implant*. (MCH nurse).

### Perceptions of maternal and child health nurses regarding women’s adherence to medical advice prior to pregnancy

The nurses reported that they did not usually advise women to seek a healthcare provider prior to pregnancy, because women only went to the healthcare facility after they were pregnant. One of the nurses explained.

*We often don’t advise women to seek a healthcare provider before pregnancy because they come to the healthcare facility already pregnant. Here, we advise women about treatment and preventing vertical transmission of HIV and contraceptive use.* (MCH nurse).

The nurses also stressed they had never received HIV positive women or couples seeking advice or counselling prior to pregnancy. They further expressed their scepticism of the acceptability of women sharing their pregnancy intentions with healthcare providers. This is because women mostly visited the healthcare facility when they were in need. One nurse expressed her view in this way:

*Even if we tell women to seek a healthcare provider before pregnancy, they might not follow this advice. Women often come to the healthcare facility when they are sick, pregnant or breastfeeding. You mostly see women because they are pregnant, and they are coming for the first antenatal care*. (MCH nurse).

Nevertheless, nurses said they knew could make a difference to HIV positive women; as one nurse explained.

*I know I can counsel HIV positive women about when to have a safe pregnancy. We first check if her CD4 allows it. She can only get pregnant if her CD4 is equal or more than 350 cells. This is the recommended CD4 level. If the CD4 is less than 350 cells, she should wait because the lower the CD4 the higher the chance of passing HIV to* [the] *infant*. (MCH nurse).

## Discussion

This study suggests women’s decision-making on pregnancy is influenced by a number of factors. These include individual perceptions, socio-cultural norms and medical advice. The findings reveal that participants value being pregnant and having children and that this is closely related to perceptions of female adulthood. The women also regarded motherhood as fundamental to female identity and status in their society. These results show a congruence with other findings in Southern Mozambique [35], as well as other sub-Saharan countries such as Botswana [36] and South Africa [37].

Perceptions of pregnancy by HIV positive women are anchored in the symbolism society places upon children [35]. Together with the pressure of male partners and families, this largely influences the decision to become pregnant. The influence of male partners and families upon pregnancy decision-making has also been documented in other studies [38, 39].

Motherhood among HIV positive women is perceived no differently to other women: it represents a way of fulfilling a woman’s role prescribed by society. As paradigms of maternity suggest [40], women are expected to marry and perform their reproductive task so as to perpetuate the lineage of their husband’s family. In the context of HIV/AIDS, becoming a mother may also offer compensation for the negative social status of a HIV woman.

In Mozambican society, where traditions and religious norms are very influential [41], children have high social and economic value [42]. Therefore, HIV positive women risk double stigmatisation if they do not have children: one related to their HIV positive status and the other related to being childless.

As studies [43] in sub-Saharan Africa have shown, childlessness may lead to stigma, rejection, and a loss of social status. However, as it has been documented in Zambia [44], participants were equally aware that a large number of children could burden their lives. Moreover, many women perceived, and were concerned about their own health-related risks as well as those of the baby during pregnancy.

This study revealed that most participants were unaware of their HIV positive status prior to pregnancy and had not complied with the medical advice recommended by the Ministry of Health of Mozambique concerning when to become pregnant. Those who did know their HIV positive status, some did not comply with antiretroviral therapy due to economic obstacles. These included, lack of money for transportation and food as well as insufficient knowledge regarding the benefits of antiretroviral therapy before pregnancy.

The correlation between the low adherence to antiretroviral therapy and economic obstacles has also been reported in Mozambique’s rural Zambézia province [21]. However, a study in the country’s Sofala province suggested food assistance provided to HIV patients had no effect on their adherence to antiretroviral therapy [45]. Nevertheless, lack of access to food is a critical barrier to consistent adherence to antiretroviral therapy. For example, in Mozambique approximately 54,7% of the population live on less than 2USD each day [46]. Unless people are able to access enough food to meet their needs, adequate adherence to antiretroviral therapy will remain a challenge.

Food insecurity as a barrier to antiretroviral therapy adherence is also a challenge in several other sub-Saharan countries. A study in Uganda [47] also determined food insecurity contributed to the lack of adherence to antiretroviral therapy. In addition, discontinuation of the therapy occurred because patients experienced that antiretroviral drugs increased their appetites leading to intolerable hunger. Moreover, the same study highlighted antiretroviral drugs side effects were greater in the absence of food. However, patients believed they should skip doses or not start on antiretroviral therapy at all, if they could not afford to buy food [47]. Similar findings were previously documented in Botswana and Tanzania [48], Zambia [49] and later in the Congo [50] as well as other sub-Saharan countries [51].

This study echoes many of the findings in the above countries which revealed how low adherence to antiretroviral therapy was related to several other significant factors: fear of HIV disclosure [50, 51]; stigma and discrimination; side effects of antiretroviral drugs [49, 52]; transportation costs; and long waiting periods at health facilities to obtain antiretroviral drugs [48, 53, 54]. A further factor was the inadequate use of modern contraceptives. The findings reveal that most participants became pregnant unintentionally. This has also been confirmed by other studies [17, 55, 56]. In general, women lack decision-making power over the use of contraception, and therefore over the appropriate time for them to get pregnant. Decisions to comply with medical advice, such as using modern contraceptives, tend to be the reserve of a woman’s male partner. This shows the extent to which women tend to rely on familial or communal norms [29] when deciding about pregnancy [57, 58].

Furthermore, the results suggest that contraceptive use and the risks of HIV-infection are less prominent than one ought to expect when it comes to decision-making about pregnancy. Following similar studies in Zambia [59] and Uganda [12], there is a pattern of HIV infection playing a minor role in family planning decisions. Most participants avoided healthcare providers because they regarded decisions about family planning as private matters; not as a consequence of insufficient knowledge. This belief may also be associated with the fact that sharing family planning intentions with healthcare providers was not a perceptible norm amongst the communities studied [29]. As numerous studies [8, 12] have shown, decisions are based more upon women’s perceptions of health, and when they feel it is necessary to have children. Consistent with other studies, [35, 60] most participants did not associate having sex with HIV reinfection, nor did they acknowledge the risk factor of mother-to-child transmission of HIV. The fulfilment of motherhood, and compliance with social norms, appeared to be more important than preventing HIV-infection through contraceptive use [61].

Finally, despite their efforts in counselling about modern contraceptive use, healthcare providers missed opportunities to refer women to healthcare facilities prior to pregnancy. A greater emphasis on referral could strengthen the relationship between healthcare providers and HIV positive women with all the attendant benefits to health this could arguably bring. Others studies [62] have also disclosed HIV positive women did not often receive sufficient support for safe conception in sub-Saharan Africa. This result is congruent with findings in Kenya [63] and Lesotho [64]. In short, the evidence suggests that healthcare providers must deal with non-compliance of women to medical advice for all the reasons cited above.

At the individual level, healthcare providers need to ensure that couples are cognizant of the benefits to consistent adherence to antiretroviral therapy while explaining its side effects. They should follow-up on patients’ experiences and advise all couples to seek medical instruction prior to pregnancy. Antiretroviral therapy should be considered a routine “medicine of the body” and the only way to ensure the health of the patients, rather than as an instrumental medication to prevent passing HIV from mother to infant. In addition, modern contraceptive use, including the use of condoms, should be explained to male partners. The study shows that when these issues are clearly explained to men, they are more inclined to accept their wives using contraceptives. However, both women and male partners should use modern contraceptives as a matter of routine, rather than a periodic measure during breastfeeding.

At the community/familial level, people should be informed about the impact of HIV, as well as the benefits of antiretroviral therapy and modern contraceptives to family planning. This information could be communicated via healthcare providers during their routine lectures to all people and when meeting patients at their clinics. Additionally, community health workers, community leaders, and non-government organisations could disseminate this information as part of their primary HIV prevention activities.

If consistent adherence to antiretroviral therapy is to be realised, a feasible strategy of antiretroviral drug distribution which does not require patients to go to the healthcare facility every month needs to be established on a national basis. This could minimise the burden of transportation costs for those who live great distances from the healthcare facility.

## Limitations

This study is subject to methodological limitations. First, the reports of pregnancy-decision making were retrospective; accuracy in recalling details may have affected the consistency of some women’s narratives in relation to their practices prior to pregnancy, and it may have been less than optimal. Secondly, the study is subject to a sample-bias because it did not access other HIV positive women who did not use the healthcare facility during the study period. Notwithstanding this, we believe that the general trajectory of the findings reported here are not significantly affected by these factors.

## Conclusion

Findings of this study reveal that HIV positive women have varying perceptions of meaning and process related to pregnancy. These are often derived from social expectations embedded in familial and/or communal norms where the community places a very high symbolic value on human reproduction. Moreover, HIV positive women lack decision-making power regarding pregnancy, and this is rooted in socio-cultural norms where perceptions of women’s role in marriage, the representation of pregnancy and reproduction are shaped by the community, rather than medical advice.

## Abbreviations

ART: Antiretroviral therapy
CRC: Child at risk clinic
FGDs: Focus group discussions
HIV/AIDS: Human immunodeficiency virus/acquired immunodeficiency syndrome
MCH: Maternal and child health nurse
PMTCT: Prevention of mother-to-child transmission
SPSS: Statistic package for Social Sciences

## Footnotes

1 CD4 cells – also known as T-cells – are types of white blood cells that protect the body from infection. When a person is infected by HIV, the virus attacks and destroys the CD4 cell of the person’s immune system. The role of CD4 cells is to protect the immune system of HIV infected people (Castilla et al., 2005). Studies (Castilla et al., 2005; Barreiro et al. 2007) have shown HIV is suppressed when CD4 count is 350 cells and above. However, the risk of transmission can be minimised, but never eliminated.

2 Charms are set of portions traditionally used as indigenous contraceptives. They are prepared by mixing the pad used on the first menstruation with plants and staffed nail’s carcass and, placing it in a closed place or burring it (Capurchande et al. 2015).

3 Serodiscordant couple is a mixed-status in which one partner is infected by HIV and the other is not.

4 Family planning card is a document issued by the healthcare facility for women who adhere to modern contraception. Patients use this card to access contraceptives at the healthcare facility.
